# Validation of ANG-1 and P-SEL as biomarkers of post-COVID-19 conditions using data from the Biobanque québécoise de la COVID-19 (BQC-19)

**DOI:** 10.1186/s12014-023-09436-7

**Published:** 2023-10-24

**Authors:** Eric Yamga, Antoine Soulé, Alain Piché, Amin Emad, Madeleine Durand, Simon Rousseau

**Affiliations:** 1 Department of Medicine, Centre Hospitalier de l’Université de Montréal, Montréal, Québec Canada; 2 Department of Electrical and Computer Engineering, McGill University, Montréal, Canada; 3 Département de Microbiologie et Infectiologie, Faculté de Médecine et des Sciences de la Santé, Université de Sherbrooke, Sherbrooke, QC Canada; 4 Centre de recherche du Centre Hospitalier de l’, Université de Montréal (CRCHUM), Montréal, Québec Canada; 5 The Meakins-Christie Laboratories at the Research Institute of the McGill University Health Centre Research Institute, Montréal, QC Canada

**Keywords:** Long COVID, Proteomics, Replication study, Biobank

## Abstract

The quest for understanding and managing the long-term effects of COVID-19, often referred to as Long COVID or post-COVID-19 condition (PCC), remains an active research area. Recent findings highlighted angiopoietin-1 (ANG-1) and p-selectin (P-SEL) as potential diagnostic markers, but validation is essential, given the inconsistency in COVID-19 biomarker studies. Leveraging the biobanque québécoise de la COVID-19 (BQC19) biobank, we analyzed the data of 249 participants. Both ANG-1 and P-SEL levels were significantly higher in patients with PCC participants compared with control subjects at 3 months using the Mann-Whitney U test. We managed to reproduce and validate the findings, emphasizing the importance of collaborative biobanking efforts in enhancing the reproducibility and credibility of Long COVID research outcomes.

Dear Editor,

We read with great interest the article recently published by Patel et al. that identified angiopoietin-1 (ANG-1) and p-selectin (P-SEL) as two diagnostic biomarkers associated with long COVID [1]. Long COVID, now formally known as post-COVID-19 condition (PCC), is diagnosed in individuals who after the acute phase of the disease experience ongoing or new symptoms three months after their initial episode, persisting for at least two months, and that cannot be explained by another condition [2]. In the referenced study, the authors identified 14 blood biomarkers of vascular transformation associated with PCC; however only ANG-1 and P-SEL were deemed essential when ranking features with Boruta feature reduction.These findings support previous data suggesting a role for endotheliopathy in the pathogenesis of PCC [3].

As encouraging as these findings are, the reproducibility of biomarkers association studies in COVID-19 has been inconsistent [4]. A recent meta-analysis of PCC highlighted the need for reliable validation studies to draw robust conclusions from these efforts [5]. Among the 18 biomarkers previously associated with PCC, the authors could only demonstrate statistical significance for six: CRP, D-dimer, LDH, leukocytes, lymphocytes, and IL-6. The association between the 12 remaining biomarkers and PASC was not statistically significant. Other biomarkers, such as ANG-1 and P-SEL, could not be validated because they were only analyzed in the aforementioned study [1]. This led some authors to suggest that independent candidate biomarkers validation should be prioritized [6].

Here, we showcase the biobanque québécoise de la COVID-19 (BQC19), a comprehensive biobank of samples and data collected from a prospective observational cohort that can be used to validate findings and generate new knowledge in patients with COVID-19-related disorders [5]. The BQC19 is a multicentric biobanking infrastructure comprising a network of 10 study sites in Québec, including clinics dedicated to the care of patients suffering from long COVID. The biobank has recruited more than 6000 SARS-CoV-2 positive and negative participants since the spring 2020. Clinical and multi-omic data are available, including genome-wide sequencing (GWS), viral genome sequencing, proteomics, serologies, metabolomics and transcriptomics, which are available for a subset of the participants. The content of the collection can be explored at the following link: https://bqc19.c3g.calculquebec.ca/.

We report the analysis of an observational cohort of 249 participants from the BQC19 for which proteomic data were available. Among these, 139 participants met the definition of PCC and 110 participants did not. PCC was operationalized as participants presenting at least one candidate symptoms at least 3 months following a positive COVID-19 PCR test. In agreement with WHO definition of PCC, the candidate symptoms included fatigue, sleep disturbance, dizziness, chest pain, arrhythmia, dyspnea, cough, abdominal pain, diarrhea, joint pain, myalgia, skin rash, anxiety and depression, headache, loss of taste or smell and brain fog. Conversely, controls were defined as participants with a positive COVID-19 PCR test who did not present any PCC associated symptoms 3 months after the acute infection. All patients in the biobank underwent periodic assessments at 3, 6, 12, 18, and 24 months in specialized post-COVID clinics. Medical professionals systematically documented the presence or absence of PCC-related symptoms for each patient.

The baseline characteristics of the study population are shown in Table 1 and the symptoms reported by PCC positive patients at 3 months of follow-up are shown in Table 2. Our participants’ enrollment date in relation to the first vaccination campaign is shown in Fig. 1.

We compared the circulating levels of the two biomarkers of interest, ANG-1 and P-SEL, as measured via SOMAmers relative fluorescent units (RFU) using the Mann-Whitney U test. Both ANG-1 and P-SEL levels were significantly higher in patients with PCC compared with control subjects at month 3 (p < 0.05; see Table 3).

This result is consistent with the study of interest but differs in the proteomic assessment method used. Patel et al.’s group used a bead-based multiplex assay (based on the Luminex ® xMAP ™ technology) that allows for direct quantification of proteins. We used the aptamer-based Somalogic platform designed as a discovery platform that measures relative concentration of proteins. The concordance of the two analyses suggests that the findings are independent of the measurement technique. Our operationalization of the PCC definition is also a limitation as we could not assess the persistent nature of the symptoms nor exclude alternative causes, as required by WHO. More broadly, defining PCC in retrospective studies remains challenging. Because of its diverse symptomatology, the reliance on self-reported symptoms, and the lack of available diagnostic tests, valid diagnostic criteria have yet to be determined. New approaches to overcome the current limitations of defining PCC cohorts include deriving dynamic PCC probability scores based on the relative prevalence of symptoms observed in both infected and non-infected participants. Those still mandate further validation [7].

As we continue to learn more about the long-term impacts of COVID-19, it is critical that we identify reliable biomarkers that can help us diagnose and potentially serve as targets to treat long-COVID. Importantly, we must also promote and develop accessible biobanks, like the BQC19, that facilitates reproducibility and enhances the validity of the findings.

**Fig. 1:**
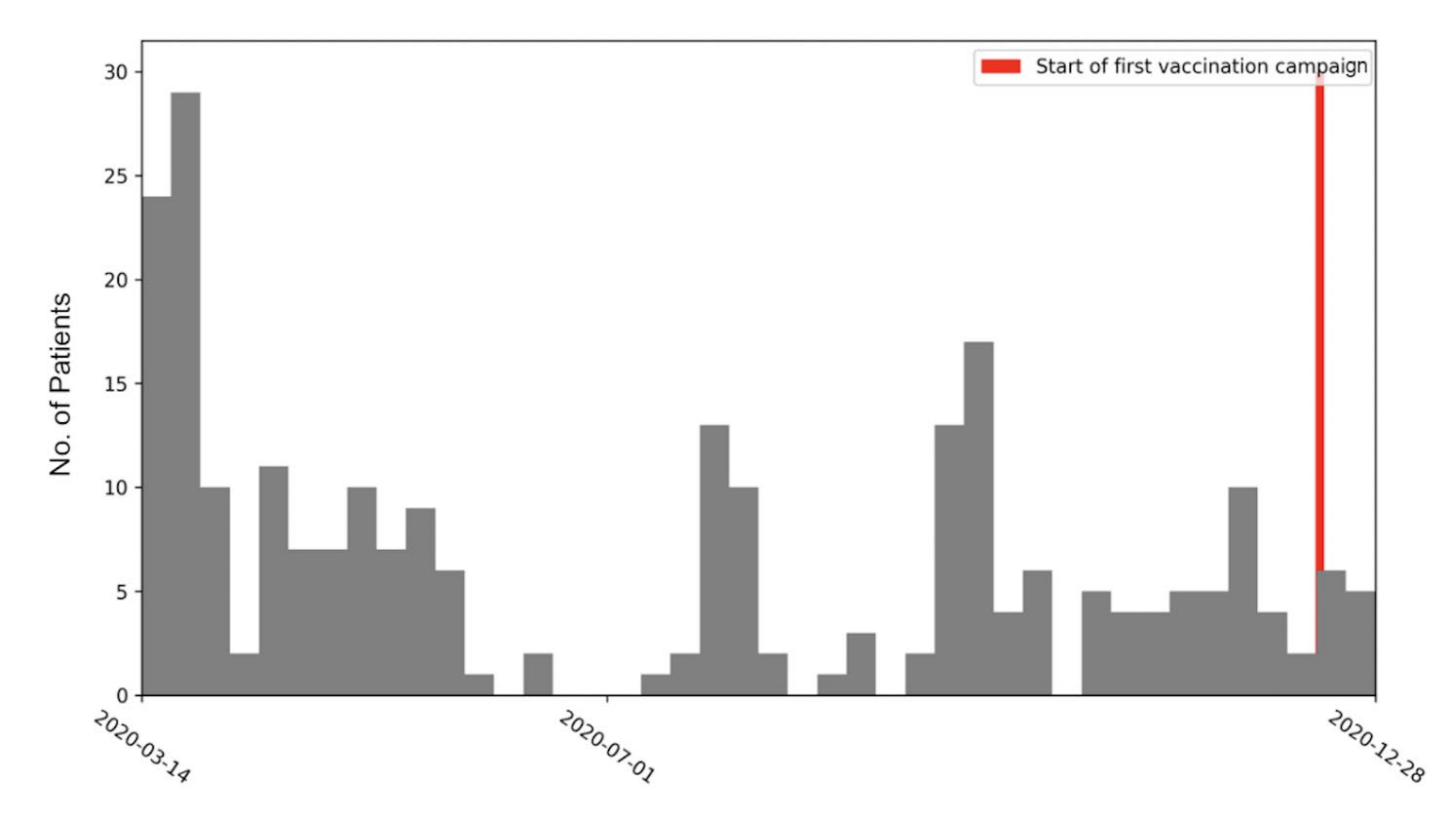
Participants weekly enrolment

*Weekly enrollment of participants over time, with the red vertical line marking the onset of the first vaccination campaign. Notably, the majority of participants joined the study prior to the initiation of vaccination efforts. Thus, vaccination did not play a significant role in the observed findings.*

**Table 1:** Baseline characteristics of our study cohort

Characteristic	PCC + (*n* = 139)	PCC - (*n* = 110)
**Age, n (%)**		
< 45	36 (25.9)	39 (35.5)
45–65	78 (56.1)	44 (40.0)
> 65	25 (18.0)	27 (24.5)
**Female sex, n (%)**	82 (59)	51 (46)
**BMI, n (%)**		
< 20	4 (2.9)	5 (4.5)
20–25	34 (24.5)	24 (21.8)
25–35	77 (55.4)	59 (53.6)
> 35	17 (12.2)	10 (9.1)
Unknown	7 (5.0)	12 (10.9)
**Hospitalization, n (%)**	(57.5)	(37.4)
**Disease severity, n (%)**		
Severe	20 (14.4)	10 (9.1)
Moderate	34 (24.5)	14 (12.7)
Mild	77 (55.4)	76 (69.1)
Unknown	8 (5.8)	10 (9.1)

Disease severity was based on the WHO COVID-19 scale

**Table 2:** Symptoms reported by PCC participants at least 3 months after the acute COVID-19 episode

	PCC+ (*n* = 139)
**Constitutional, n (%)**	
Fatigue	69 (49.7)
Sleep disturbance	3 (1.7)
Dizziness	9 (6.5)
**Cardiac, n (%)**	
Chest pain	12 (8.6)
Arrhythmia	1 (0.7)
**Respiratory, n (%)**	
Dyspnea	46 (33.1)
Cough	21 (15.1)
**Gastrointestinal, n (%)**	
Abdominal pain	12 (8.6)
Diarrhea	7 (5.0)
**Musculoskeletal, n (%)**	
Joint pain	28 (20.1)
Myalgia	19 (13.7)
Skin rash	9 (6.5)
**Neurological, n (%)**	
Anxiety and depression	57 (41.0)
Headache	22 (15.8)
Loss of taste /smell	29 (20.9)
Brain fog	6 (4.3)

**Table 3:** Pairwise comparisons of vascular biomarkers

Target vascular biomarker	Sampling time	PCC – (*n* = 110)	PCC + (*n* = 139)	Mann-Whitney U test (two-sided)	
		Median RFU		U	p-value
P-Selectin	3 months	-0.32	0.05	6267	0.0147
ANG-1	3 months	-0.66	-0.42	6525	0.0474

## Data Availability

Access to the BQC19 biobank is possible through the BQC19 access committee.

## Abbreviations

ANG-1: Angiopoietin-1
BQC19: Biobanque québécoise de la COVID-19
P-SEL: P-selectin
PCC: Post-COVID-19 condition
RFU: Relative fluorescent units
